# Novel three-sinus enlargement technique for supravalvular aortic stenosis without aortic transection

**DOI:** 10.1186/s13019-016-0403-5

**Published:** 2016-01-16

**Authors:** Shinya Yokoyama, Hisao Nagato, Yuichi Yoshida, Shigeo Nagasaka, Kozo Kaneda, Noboru Nishiwaki

**Affiliations:** Department of Cardiovascular Surgery, Kinki University School of Medicine Nara Hospital, 1248-1, Otoda-cho, Ikoma, Nara, 630-0293 Japan

**Keywords:** Supravalvular aortic stenosis, Aortic valve stenosis, Three-sinus enlargement, Infant

## Abstract

**Background:**

Although repair of a supravalvular aortic stenosis (SVAS) can be performed with low mortality rates, surgery for the complex form of SVAS continues to be associated with a high incidence of residual stenosis.

**Case presentation:**

The patient was referred to our hospital at 1 month of age and was diagnosed with aortic valve stenosis (AS) by using echocardiography. Cardiac catheterization revealed moderate AS, and subsequent left ventriculography revealed discrete stenosis of the sino-tubular junction and a narrowed proximal ascending aorta. We performed a reconstructive operation for such heart defects involving novel three-sinus and ascending aorta enlargement without aortic root transection in a 6-month-old boy.

**Conclusion:**

Our novel three-sinus enlargement technique is suitable for treating each type of SVAS and is a useful method for a baby particularly less than 10 kg without disturbing the growth of the ascending aorta.

## Background

Although repair of a SVAS can be performed with low mortality rates, surgery for the complex form of SVAS, including repair of a diffuse narrow ascending aorta or single patch repair, continues to be associated with a high incidence of residual stenosis. Here, we report a case wherein successful novel three-sinus and ascending aorta enlargement was performed in an infant. The new procedure involves three-dimensional combination of three patches without aortic root transection.

## Case presentation

**Fig. 1:**
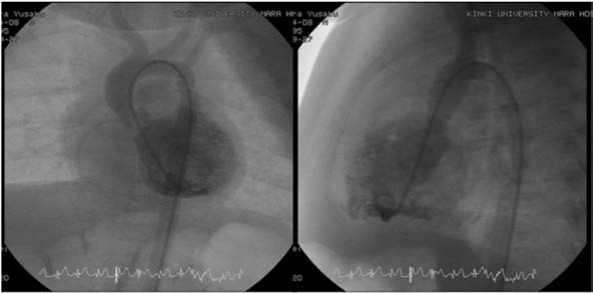
Preoperative left ventriculography showed discrete stenosis of the sino-tubular junction and a narrowed proximal ascending aorta

The patient was a 6-month-old male infant (weight, 5.9 kg) diagnosed with aortic valve stenosis by echocardiography. Cardiac catheterization revealed moderate aortic valve stenosis due to thickened tricuspid valve, with left ventricular pressure of 143/- mmHg (end-diastolic pressure 11 mmHg) and an ascending aortic pressure of 83/38 mmHg (mean, 57 mmHg). Subsequent left ventriculography revealed discrete stenosis of the sino-tubular junction and a narrowed proximal ascending aorta [Fig. 1].

The operation was performed at 6 months of age. After median sternotomy, a 30-mm square piece of autologous pericardium was harvested and treated with 0.6 % glutaraldehyde solution. Subsequent morphological inspection of the outer structure was performed, and features nearly identical to those at the preoperative diagnosis were noted. Cardiopulmonary bypass was established with ascending aortic and bicaval cannulations, aortic cross clamping was performed and cardiac arrest was achieved with cold crystalloid cardioplegia.

**Fig. 2:**
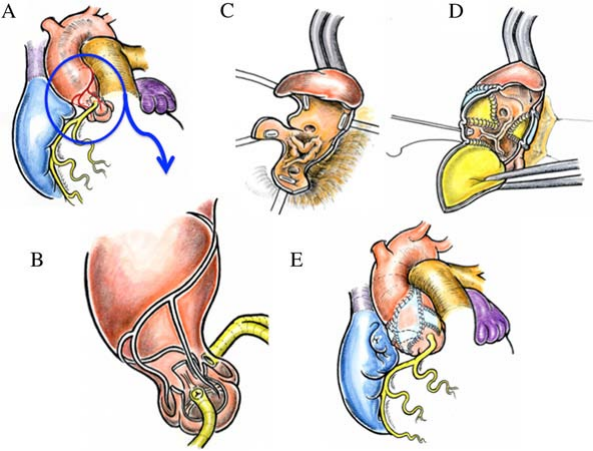
Schematic diagram of the surgical technique: **a** The illustration shows the outer appearance before operation. **b** The incision line. **c** The ascending aorta is incised along the incision line. **d** The second and third incisions were enlarged using a trimmed ellipse patch respectively and the initial oblique incision was enlarged using fontanel shaped patch. **e** The illustration shows the outer appearance after operation

**Fig. 3:**
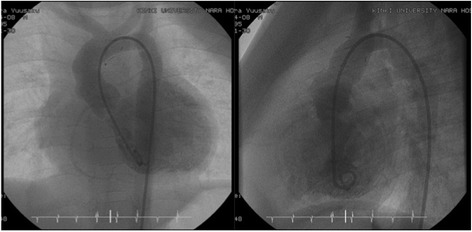
Postoperative left ventriculography at 13 months after operation showed no stenosis in the ascending aorta and maintained of symmetry of the ascending aorta

First, an oblique incision was made on the anterior wall of ascending aorta. The proximal end of the incision was at the center of the non-coronary sinus. The second incision began within the lower third of the first incision line and was placed 2–3 mm to right of the left coronary artery orifice. Because the left main trunk originated from the center of the left sinus and turned to the left, this incision avoided damage to the left coronary artery orifice. This incision and patch closure resembles the Manouguian root enlargement. Then the third aortic incision began within the upper third of the first incision line and was placed 2–3 mm to the left of the right coronary artery orifice. Because the right coronary artery originated from the center of the right sinus and turned to the right, the third incision avoided damage to the right coronary artery orifice. This incision and patch closure resembles the Konno root enlargement. After aortic valvoplasty with three directional commissurotomy and leaflet slicing, ascending aortoplasty was performed using the novel three-sinus enlargement technique. The second and third incisions were enlarged using a trimmed half-ellipse composite patch, prepared using autologous pericardium treated with 0.6 % glutaraldehyde solution and Dacron velour fabric. Finally, the initial aortic oblique incision was enlarged using a fontanel-shaped composite patch attached with a single running suture [Fig. 2]. Cardiac catheterization was performed at 13 months after the procedure, and no stenosis in the left ventricular outflow tract was observed. The pressure gradient across the aortic annulus was 10 mmHg or less [Fig. 3].

## Discussion

Although the surgical strategy for SVAS has been established, complications such as aortic valve stenosis, diffuse ascending aortic stenosis, aortic coarctation, and coronary ostial stenosis impact surgical outcome [1]. Maintaining of symmetrical proportions of the ascending aorta, sufficient aortic diameter, and no residual stenosis in the left ventricular outflow tract is crucial for successful SVAS restoration.

Although the Myers method allows for repair without patches, diffuse types of SVAS are not effectively repaired by the method [2]. Single or two-sinus enlargements using one patch result in an asymmetrical aorta [3, 4]. Furthermore, the traditional three-sinus enlargements using multiple patches require aortic root transection [5]. Aortic root transection performed during infancy can cause aortic restenosis in the future. The operative technique had not been improved for a long time. We therefore developed a new method without aortic root transection to avoid aortic restenosis.

Here, we present a novel three-sinus enlargement technique that did not require aortic root transection. The chief advantage of this method is that the three-sinus enlargement can be performed while maintaining an aortic continuity. This method is therefore believed to prevent residual aortic stenosis or future aortic restenosis owing to distortion of the reanastomotic suture line. In addition, the arrangement of the incisions is very important, and these incisions should be made with special care. If the incisions were made on the aortic wall near the coronary orifice, negative outcomes would result. Therefore, we referred to articles on aortic valve annulus enlargement techniques when creating the new arrangement of the incisions [6, 7].

The first oblique incision is an important element of the enlargement procedure for the narrow ascending aorta. The ascending aorta was incised in a spiral pattern, and patch enlargement of this incision allowed three-dimensional enlargement of the narrow aorta without aortic root transection. To obtain sufficient enlargement after repairing a long narrow ascending aorta, conventional multiple patch methods required the use of some other patches [8]. However, the present method dose not requires additional patches to repair a long narrow ascending aorta and is therefore more effective for treating diffuse-type SVAS.

## Conclusions

We report a successful novel three-sinus and ascending aorta enlargement without aortic root transection in a 6-month-old boy with SVAS and AS. Our novel three-sinus enlargement technique is suitable for treating each type of SVAS and is a useful method for a baby particularly less than 10 kg without disturbing the growth of the ascending aorta.

### Consent

Written informed consent was obtained from the patient for publication of this Case report and any accompanying images. A copy of written consent is available for review by the Editor-in-Chief of this journal.

## Abbreviations

SVAS: Supravalvular aortic stenosis
AS: Aortic valve stenosis
